# Relationship between salivary levels of interleukin‐8 and HbA1c in patients with type 2 diabetes

**DOI:** 10.1002/edm2.455

**Published:** 2023-09-29

**Authors:** Masoomeh Shirzaiy, Zohreh Dalirsani, Payam Peymankar, Mahboobeh Taherizadeh

**Affiliations:** ^1^ Oral and Dental Disease Research Center Zahedan University of Medical Science Zahedan Iran; ^2^ Oral and Maxillofacial Diseases Research Center Mashhad University of Medical Sciences Mashhad Iran; ^3^ Oral and Dental Disease Research Center Zahedan University of Medical Science Zahedan Iran; ^4^ Faculty of Health Mashhad University of Medical Sciences Mashhad Iran

**Keywords:** chemokines, diabetes mellitus, HbA1c, interleukin‐8, saliva, type 2

## Abstract

**Introduction:**

Diabetes mellitus is a metabolic disease, which genetic and environmental factors play a role in its pathogenesis. Cytokines as important elements in the immune system have diverse expressions in different individuals and societies and are effective in the pathogenesis of diabetes. This study investigated the relationship between blood sugar control and salivary levels of interleukin‐8 (IL‐8) in patients with type 2 diabetes.

**Methods:**

This cross‐sectional study was conducted on 73 subjects (35 diabetic and 38 healthy individuals). Unstimulated saliva samples were collected and the correlation between IL‐8, as an inflammatory marker and HbA1c (Haemoglobin A1C) was studied.

**Results:**

The levels of IL‐8 and HbA1c were significantly higher in the patient group than control group (*p* < .001, *p* < .001, respectively). There was not any relationship between salivary IL‐8 levels and glycemic control levels (*p* = .629). Also, there was no remarkable difference between men and women in terms of the levels of IL‐8 and HbA1c saliva (*p* = .524, *p* = .998, respectively).

**Conclusion:**

Although the salivary IL‐8 levels were higher in the diabetic patients, blood sugar control did not significantly affect cytokine concentrations. Increased salivary levels of IL‐8 in patients with type 2 diabetes could be a basis for risk assessment, prevention and treatment of diabetes‐related complications.

## Graphical abstract

Salivary levels of interleukin‐8 were higher in the diabetic patients than in the healthy subjects. There was not any association between salivary levels of IL‐8 and blood sugar control levels (HbA1c). No correlation between salivary IL‐8 levels and age in the diabetic subjects was observed. There was not any significant deference between the women and men regarding to salivary IL‐8 and HbA1c.

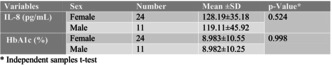

## 1 INTRODUCTION

Diabetes is one of the most prevalent chronic endocrine diseases and leading causes of mortality in the world. There is a direct relationship between poor blood sugar control and complications associated with diabetes, such as cardiovascular diseases, retinopathy and nephropathy. Therefore, and good glycemic level control can delay the diabetes complications.^1^, ^2^

Type 2 diabetes accounts for 90%–95% of all diabetic cases. Studies have shown that several factors, such as obesity, lipotoxicity, chronic inflammation and oxidative stress are involved in the pathophysiology of the diabetes.^3^ Increased blood sugar levels leads to produce more reactive oxygen species and induces peroxidation of fatty acids in the cells, consequently affects the survival of natural cells.^3^, ^4^

Cytokines are important elements in the immune system and are involved in the pathogenesis of inflammatory disease. During inflammatory and pathologic processes, these markers are produced by different cells, such as monocytes, macrophages, T lymphocytes, neutrophils and endothelial cells. They play a role in chemotaxis and activation of neutrophils, T lymphocytes, and basophils, as well as in the pathogenesis of type 2 diabetes and atherosclerosis.^5^, ^6^, ^7^, ^8^

Interleukin‐8 (IL‐8) levels could increase 10‐ to 100‐fold above normal range in response to other inflammatory cytokines, such as IL‐1 and tumour necrosis factor‐α (TNF‐α), as well as cellular stress and viral and bacterial process.^7^ The IL‐8 levels were assessed in previous studies. Zozuliñska et al. reported that the serum level of IL‐8 was significantly higher in diabetic patients than in non‐diabetics subjects.^9^ It seems that in high glucose concentration, the binding of monocytes to endothelial cells in diabetic patients leads to the production of IL‐8.^10^ Subsequently, an increase in serum level of IL‐8 causes inflammatory reactions in diabetic patients. It is a vicious cycle, in which increased levels of IL‐8 and glucose are related.^8^

Dakovic et al. reported that the salivary IL‐8 levels were higher in children with type 1 diabetes than in healthy children, and was an effective cause of microangiopathy and macroangiopathy, as well as the pathogenesis of atherosclerosis.^11^

This study was conducted to investigate any correlation between salivary IL‐8 levels and glycemic control level by considering the role of inflammatory mediators in the pathogenesis and complications of diabetes and several conflicting results in the literature.

## 2 METHODS AND MATERIALS

### 2.1 Subjects selection

In this cross‐sectional study, diabetic persons were selected from diabetic patients (type 2), attending diabetes clinic in Boo‐Ali hospital, Zahedan, Iran, during 2020.

The inclusion criteria for the case group were the following:

1. A five‐year history of type 2 diabetes,
2. They had a fasting blood sugar ≥126 mg/dL or 2‐h postprandial blood sugar level ≥ 200 mg/dL,

The exclusion criteria were the following:

1. Having other systemic diseases, such as Sjogren's syndrome,
2. History of radiotherapy,
3. Tobacco smoking or/and addiction to alcohol,
4. History of periodontitis,
5. Using vitamin supplements or antioxidants in the last 3 months.^4^

Among the persons referred to Zahedan Faculty of Dentistry, for usual dental treatment, 38 individuals without any systemic disease or chronic use of any medicine or supplement were included in the study as the controls. The same exclusion criteria for the case group were applied to the controls.

### 2.2 Evaluating study parameters

After obtaining their informed written consent, all subjects were referred to the laboratory for evaluation of FBS (Fasting blood sugar) and HbA1c. The Biosystems Kit (Spain) was used to assess HbA1c in compliance with the faculty protocol. To assess the salivary levels of IL‐8, unstimulated saliva specimens were collected between 9 and 11 a.m., using the spitting method. The participants were asked to not eating, drinking and brushing at least 90 min before collecting their saliva. They were asked to spit their saliva into one 50 mL test tube every 60 s for 2–5 min.^12^ All specimens were delivered to the laboratory and kept at −80°C to prevent salivary protein degradation until the test time.^12^

Glycemic control level was categorized as follows: Good control (HbA1C < 8.0%), poor control (8.0 ≥ HbA1C% ≤ 10.0) and very poor control (HbA1C > 10.0%).^13^

#### 2.2.1 Ethical approval

This study was approved by the Ethics Committee of Zahedan University of Medical Sciences with the code of IR.ZAUMS.REC.1399.376.

### 2.3 Statistical analyses

The collected data were inputted into SPSS (ver.21) for statistical analyses.

Kolmogorov–Smirnov normality test was used to assess normal distribution of the quantitative variables. The independent *t*‐test was applied to compare quantitative variables with normal distribution. In case of abnormal distribution, non‐parametrical test such as Mann–Whitney *U* test was used. Also, chi‐squared test were used to analyse qualitative variables between two groups. Pearson's correlation coefficient test was used to assess the correlation between the study variables. The significance level was set at 0.05.

## 3 RESULTS

### 3.1 Demographic data

In this study, 35 patients with type 2 diabetes (11 men and 24 women with the mean age of 47.74 ± 8.73 years) and 38 healthy people (19 men and 19 women with the mean age of 47.58 ± 8.72 years) were evaluated.

The Mann–Whitney and chi‐squared tests showed that two groups were statistically matched according to the age and sex (*p*‐value = .881 and *p*‐value = .107, respectively). The mean duration of their diabetes was 8.2 ± 4.3 years in the case group.

### 3.2 Assessment of HbA1c and IL‐8

The mean HbA1c in the diabetics (8.98 ± 1.44%) was significantly higher than in the healthy subjects (4.2 ± 1.35%) (*p* < .001). According to Table 1, the salivary levels of IL‐8 were higher in the case group than in the control group (*p* < .001). Table 2 revealed the salivary levels of IL‐8 in the case group according to the glycemic control levels (*p* = .479).

**TABLE 1:** Salivary IL‐8 levels in the studied groups.

Variable	Group	Number	Mean ± SD	*p*‐value^a^
IL.8 (pg/mL)	Diabetic	35	125.34 ± 38.41	<.001
	Control	38	80.63 ± 34.76	
HbA1c (%)	Diabetic	35	8.98 ± 1.44%	<.001
	Control	38	4.2 ± 1.35%	

^a^ Independent samples *t*‐test.

**TABLE 2:** Salivary IL‐8 levels in the diabetic patients considering to glycemic control levels (HbA1c).

HbA1c	Number	IL‐8 (Mean ± SD) (pg/mL)	*p*‐value^a^
Good (<8.0%)	10	132.74 ± 45.62	.479
Poor or very poor (≥8%)	25	122.39 ± 35.72	
Total	35	125.34 ± 38.41	

^a^ Independent samples *t*‐test.

Also, among diabetic subjects, there was not any significant difference between the women and men regarding to salivary IL‐8 and HbA1c (*p* = .524 and *p* = .998, respectively) (Table 3).

**TABLE 3:** The comparison of IL‐8 and HbA1c levels between female and male diabetic patients.

Variables	Sex	Number	Mean ± SD	*p*‐value^a^
IL‐8 (pg/mL)	Female	24	128.19 ± 35.18	.524
	Male	11	119.11 ± 45.92	
HbA1c (%)	Female	24	8.983 ± 10.55	.998
	Male	11	8.982 ± 10.25	

^a^ Independent samples *t*‐test.

Moreover, Pearson's correlation coefficient test showed no correlation between salivary IL‐8 levels and age in the diabetic subjects (*p* = .629) (Table 4). As well as, there was not any remarkable relationship between HbA1c and IL‐8 in the diabetic patients (*p* = .749) (Table 4).

**TABLE 4:** Correlation between IL‐8 with age and HbA1c in the diabetic patients.

Variables	Number	Pearson's correlation coefficient test(r)	*p*‐value
IL‐8 levels	35	0.085	.629
Age			
IL‐8 levels	35	0.056	.749
HbA1c			

**FIGURE 1:**
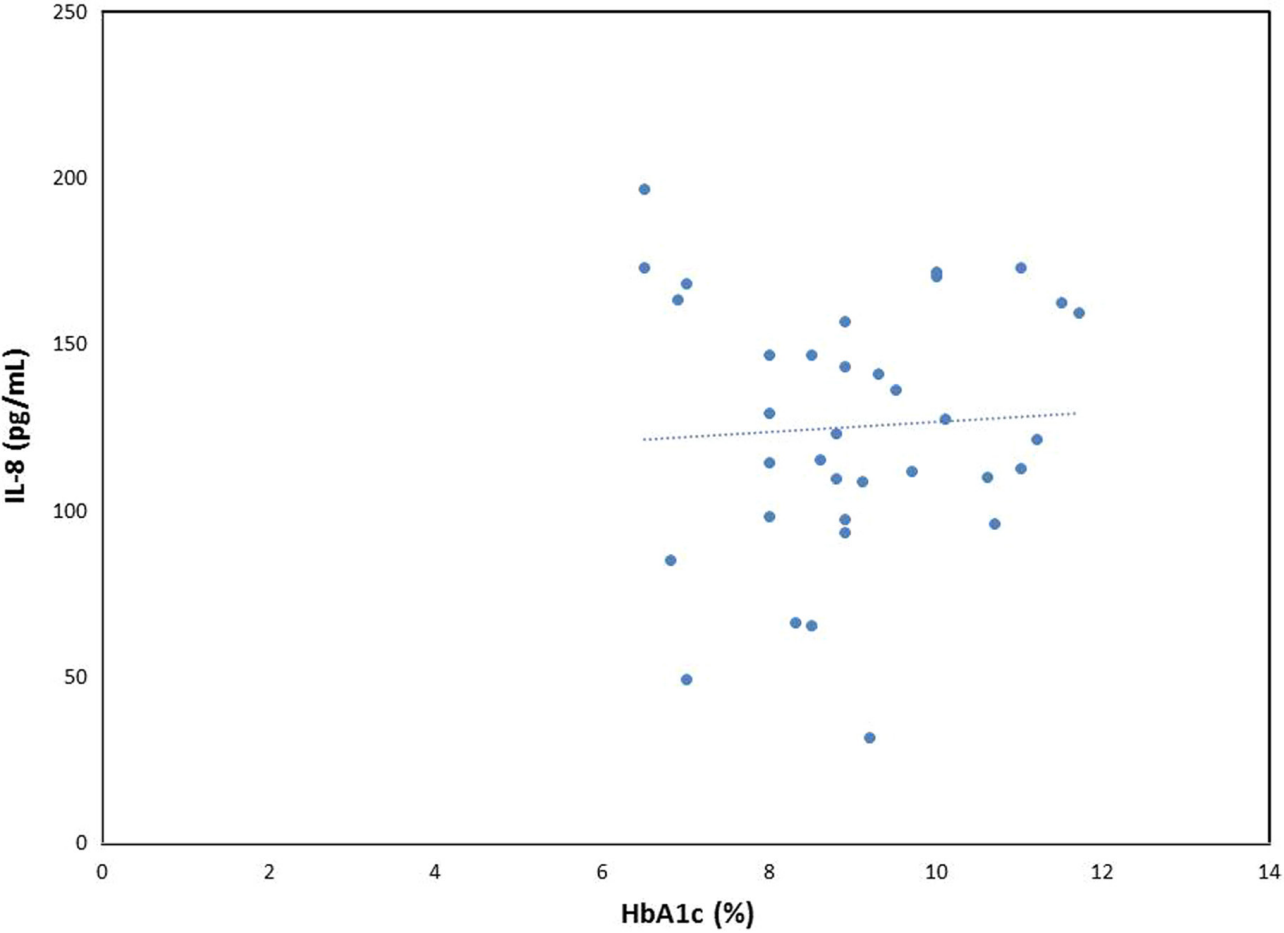
Scatter plot of distribution of patients' HbA1c according to IL‐8 levels.

Figure 1 demonstrates the distribution of patients' HbA1c according to interleukin‐8 concentration, which indicates that there is no relationship between HbA1c percentage and IL‐8 concentration.

## 4 DISCUSSION

Diabetes mellitus is a metabolic disease caused by impaired production of insulin or resistance to it and is characterized by the abnormal metabolism of glucose, proteins and lipids.^14^ Non‐insulin‐dependent (type 2) diabetes mellitus, which is the most prevalent type of diabetes, is an inflammatory disease, where inflammatory cytokines have play a role in its pathogenesis.^15^

Cytokines are largely produced by macrophages and stimulated lymphocytes, and are involved in acute inflammatory responses.^16^, ^17^ IL‐8 is one of the cytokines and is produced by monocytes, macrophages, T lymphocytes, neutrophils, endothelial, etc. during inflammatory and pathologic processes and may have a role in the pathogenesis of type 2 diabetes, as well as atherosclerosis.^8^

In the present study, the salivary level of IL‐8 was significantly higher in the patients with type 2 diabetes than in the healthy persons. Dakovic et al. also reported that the salivary concentration of IL‐8 was remarkably higher in the diabetic people than in the control subjects.^11^

Previous studies demonstrated an increase in serum IL‐8 levels in diabetic patients.^8^, ^9^, ^15^, ^18^ It is suggested that the serum level of IL‐8 can be used as a predictive factor of diabetes‐related microvascular and macrovascular diseases, especially in high‐risk patients.^15^

In contrast, Longo’ s study did not show any significant difference between studied groups (including diabetic patients with and without periodontitis and healthy subjects without periodontitis) according to the sera levels of inflammatory markers such as IL‐6 and IL‐8.^19^

Also, Tavangar et al. demonstrated higher IL‐8 serum level in diabetic patients with oral lichen planus (OLP) than its concentration in diabetes without OLP as well as control subjects. Also, the mean concentrations of FBS and 2 h postprandial blood sugar (2 h PP) in the diabetic with OLP patients was higher than healthy subjects; however, this difference was not significant between the diabetic patients without OLP and healthy patients.^16^ Although, the serum level of this factor was higher in diabetic people without OLP lesions than healthy individuals; this difference was not statistically significant,^16^ which was inconsistent with the findings of the present study. Also, Katherine Esposito, et al. reported a remarkable correlation between FBS and IL‐8 concentration in the diabetic patients.^18^

Studies on different body fluids, the differences in the dietary pattern, genetic diversity and ethnic characteristics in different geographic regions could explain these contradictory results.

Since the serum elements also secreted into the saliva, changes in the serum levels of IL‐8 can affect the salivary concentration of this factor. Recent studies have shown that the IL‐8 is secreted by adipocytes; furthermore, the circulating IL‐8 levels in obese people with diabetes are significantly higher than in normal‐weight patients, indicating a positive correlation between the circulating level of IL‐8 and body mass index (BMI).^20^ In the present study, the participants' BMI was within the normal range.

The present study was conducted on the diabetic patients without any periodontal problems. Some studies were evaluated the IL‐8 levels, as an inflammatory element, in diabetic individuals with periodontitis, although no association was reported between periodontitis and IL‐8 level in diabetic patients.^21^, ^22^

Since glucose binds to haemoglobin and other proteins, HbA1c is a good indicator of long‐term blood sugar control,^23^ so some studies evaluated its relationship with cytokines levels.^24^, ^25^

In the present study, there was not any correlation between the glycemic control levels (based on HbA1c) and IL‐8 concentration. Linhartova also did not observe a specific relationship between the IL‐8 levels and HbA1c.^24^

Nevertheless, Lappin et al. reported a remarkable relationship between plasma IL‐8 and glycemic control levels in type 1 diabetic patients.^25^ However, in their study, type 1 diabetic patients with periodontitis were investigated, while the present study was conducted on type 2 diabetic patients without periodontitis, which could explain this difference. Moreover, a linear correlation between HbA1c and IL‐8 levels was observed by Cimini et al.; although their study was performed on subjects' serum.^8^

IL‐8 is an inflammatory chemokine, that is largely involved in the systemic immunity, infiltration of macrophages and activation of lipid tissues and may have an important role in the pathogenesis and complications of type 2 diabetes.^22^, ^26^, ^27^

However, the role of IL‐8 in the pathogenesis of diabetes is still controversial, it seems that at high glucose levels, monocytes bound to endothelial cells and produce IL‐8,^10^ subsequently increasing IL‐8 concentration accelerates inflammatory reactions in diabetic patients.

Some studies suggested that the immuno‐biochemical markers can be used as a predictor of diabetes and their complications.^28^, ^29^ If the results be confirmed by comprehensive studies, the assessment of salivary samples, especially in high‐risk persons can be a non‐invasive alternative method for beneficial screening and a good substitute for traditional techniques, such as serum collection.

## 5 CONCLUSION

An increase in salivary IL‐8 levels was found in the type 2 diabetic patients; however, the level of glycemic control did not show any remarkable effect on this cytokine. Assessment of salivary inflammatory elements in diabetic patients could be a suggestive method for screening patients and evaluating the process of diabetes treatment.

## AUTHOR CONTRIBUTIONS

**Masoomeh Shirzaiy:** Conceptualization (lead); funding acquisition (equal); investigation (equal); methodology (equal); project administration (equal); supervision (equal); writing – original draft (equal). **Zohreh Dalirsani:** Investigation (equal); supervision (equal); writing – original draft (equal); writing – review and editing (lead). **Payam Peymankar:** Data curation (equal); methodology (equal); resources (equal); software (equal); validation (equal); writing – original draft (equal). **Mahboobeh Taherizadeh:** Data curation (equal); formal analysis (lead); methodology (equal); software (lead); validation (equal); visualization (equal); writing – original draft (equal).

## CONFLICT OF INTEREST STATEMENT

The Authors declare that there is no conflict of interests.

## Data Availability

The data that support the findings of this study are available on request from the corresponding author. The data are not publicly available due to privacy or ethical restrictions.
